# Neurochemical Changes and c-Fos Mapping in the Brain after Carisbamate Treatment of Rats Subjected to Lithium–Pilocarpine-Induced Status Epilepticus

**DOI:** 10.3390/ph10040085

**Published:** 2017-11-01

**Authors:** José Eduardo Marques-Carneiro, Astrid Nehlig, Jean-Christophe Cassel, Eduardo Ferreira Castro-Neto, Julia Julie Litzahn, Anne Pereira de Vasconcelos, Maria da Graça Naffah-Mazacoratti, Maria José da Silva Fernandes

**Affiliations:** 1 Departamento de Neurologia e Neurocirurgia, Disciplina Neurociência, Universidade Federal de São Paulo, São Paulo 04021-001, Brazil; edumarques83@gmail.com (J.E.M.-C.); eduardo_f.castro@hotmail.com (E.F.C.-N.); julie.litzahn@gmail.com (J.J.L.); naffahmazzacoratti@gmail.com (M.d.G.N.-M.); 2 Unistra, Laboratoire de Neurosciences Cognitives et Adaptatives—Université de Strasbourg, Faculté de Psychologie, 67000 Strasbourg, France; jcassel@unistra.fr (J.-C.C.); pereira@unistra.fr (A.P.d.V.); 3 Centre National de la Recherche Scientifique (CNRS), UMR 7364, LNCA, 12 rue Goethe, 67000 Strasbourg, France; 4 Institut National de la Santé et de la Recherche Médicale (INSERM-U 1129)—Infantile Epilepsies and Brain Plasticity, 75654 Paris, France; nehliga@unistra.fr; 5 Université Paris Descartes, Sorbonne Paris Cité, 91190 Gif-sur-Yvette, France

**Keywords:** carisbamate 1, temporal-lobe epilepsy 2, brain activity 3

## Abstract

The administration of lithium–pilocarpine (LiPilo) in adult rats is a validated model reproducing the main clinical and neuropathological features of temporal lobe epilepsy (TLE). Previous studies have shown that carisbamate (CRS) has the property of modifying epileptogenesis in this model. When treated with CRS, about 50% of rats undergoing LiPilo status epilepticus (SE) develop non-convulsive seizures (NCS) instead of convulsive ones (commonly observed in TLE). The goal of this work was to determine some of the early changes that occur after CRS administration, as they could be involved in the insult- and epileptogenesis-modifying effects of CRS. Thus, we performed high-performance liquid chromatography (HPLC) to quantify levels of amino acids and monoamines, and c-Fos immunohistochemical labeling to map cerebral activation during seizures. Comparing rats treated one hour after SE onset with saline (CT), CRS, or diazepam (DZP), HPLC showed that 4 h after SE onset, dopamine (DA), norepinephrine (NE), and GABA levels were normal, whereas serotonin levels were increased. Using c-Fos labeling, we demonstrated increased activity in thalamic mediodorsal (MD) and laterodorsal (LD) nuclei in rats treated with CRS. In summary, at early times, CRS seems to modulate excitability by acting on some monoamine levels and increasing activity of MD and LD thalamic nuclei, suggesting a possible involvement of these nuclei in insult- and/or epileptogenesis-modifying effects of CRS.

## 1. Introduction

Temporal lobe epilepsy (TLE) is characterized by recurrent spontaneous convulsive seizures [1] that are associated with significant neuronal loss and morphological alterations affecting mainly mesial temporal structures such as the hippocampal formation and amygdala [1,2,3]. The pilocarpine-induced epilepsy model reproduces the main clinical and pathophysiological features of human TLE, i.e., hippocampal sclerosis, cell dispersion in the dentate gyrus, mossy fiber sprouting, and gliosis [4,5]. In this model, an initial phase of status epilepticus (SE) is followed by a latent period lasting 7–44 days, leading to the occurrence of spontaneous recurrent motor seizures [4]. In addition, this model also reproduces significant neuronal damage in the mesial structures of the brain [6].

Despite numerous commercially available antiepileptic drugs, approximately 20–30% of patients with TLE present refractory seizures [7]. Experimental studies have shown that a new drug, carisbamate (CRS, RWJ-333369; S-2-*O*-carbamoyl-1-o-chlorophenyl-ethanol) possesses the property of modifying epileptogenesis in the TLE model induced by lithium–pilocarpine (LiPilo) [8,9,10]. When injected at one hour after SE onset in rats, CRS induces widespread neuroprotection and alters the development of TLE in approximately 50% of the rats, leading to non-convulsive absence-like seizures (NCS) instead of the complex partial seizures of the LiPilo model [10]. In these animals, NCS are characterized by behavioral arrest accompanied by bilateral synchronous SWDs [11,12]. Interestingly, most rats treated with CRS that later develop NCS show a reduction of epileptiform events during SE at 2–3 h after CRS administration, i.e., at 4–5 h after SE onset [10].

**Figure 1:**
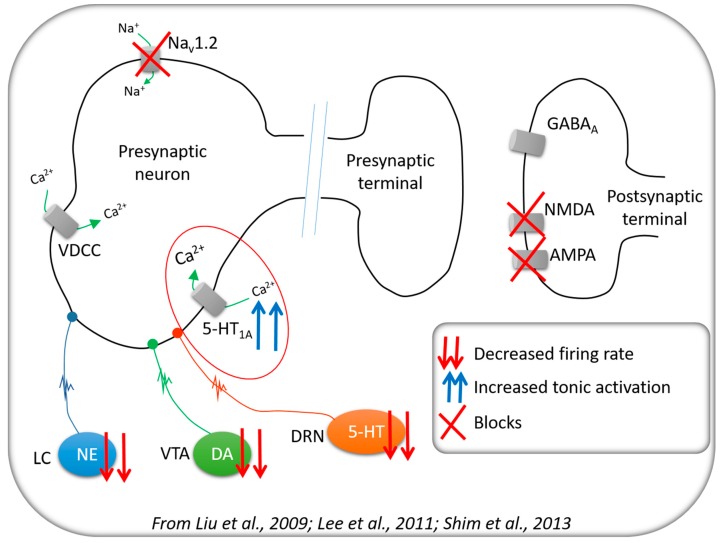
Summary of the main systems modulated by carisbamate. Legend: NE—norepinephrine; DA—dopamine; 5-HT—serotonin; VDCC—voltage-dependent calcium channel; LC—locus coeruleus; VTA—ventral tegmental area; DRN—dorsal raphe nucleus.

Previous studies have shown that CRS may modulate neuronal excitability by inhibiting voltage-gated sodium channels, thereby reducing action potential discharges [13]. In addition, CRS reduces glutamatergic transmission by inhibiting AMPA and NMDA excitatory post-synaptic potentials [14]. More recently, it was reported that CRS increases the tonic activation of somatodendritic 5-HT_1A_ serotonergic receptors, leading to the inhibition of pyramidal neurons in the hippocampus. Moreover, CRS modulates noradrenergic and dopaminergic systems [15]. Some of the mechanisms modulated by CRS are illustrated in Figure 1.

Based on previous studies, we can hypothesize that CRS reduces neuronal excitability by acting synergistically on several neurotransmitter systems and pathways. Supporting these data, we recently reported [16] that CRS-NCS rats display alterations in proteins related to cellular respiration and energy production processes, which may also impact on neuronal excitability. Among altered proteins we observed a reduction of alpha-synuclein in rats treated with CRS and an increase of the same protein in CRS-treated animals displaying NCS instead of convulsive seizures [16]. A recent hypothesis suggests that epigenetic alterations in some proteins such as alpha-synuclein may be associated with the formation of new ion channels that may disrupt membrane conductance and underlie the change in epilepsy type induced by CRS treatment [17].

In the present study, we were interested in verifying whether the epilepsy-modifying effect of CRS could be related to early changes in the amino acid and monoamine neurotransmission balance, and whether CRS could act on brain regions related to seizure spread and control. We expected to identify potential molecular mechanisms underlying the decrease in convulsive activity, which later results in neuroprotection [10]. We chose to more specifically study hippocampus and parahippocampal cortices since they are key structures in TLE, as well as the thalamus, which is involved in both TLE and absence seizures [12,18]. The studies were performed at 4 h after SE onset, i.e., when a reduction of epileptiform events has been observed [10]. With this study, we aimed at improving our knowledge on the early functional changes underlying the disease and epileptogenesis-modifying effects of CRS.

## 2. Results

### 2.1. Monoamine and Amino Acid Quantification

**Figure 2:**
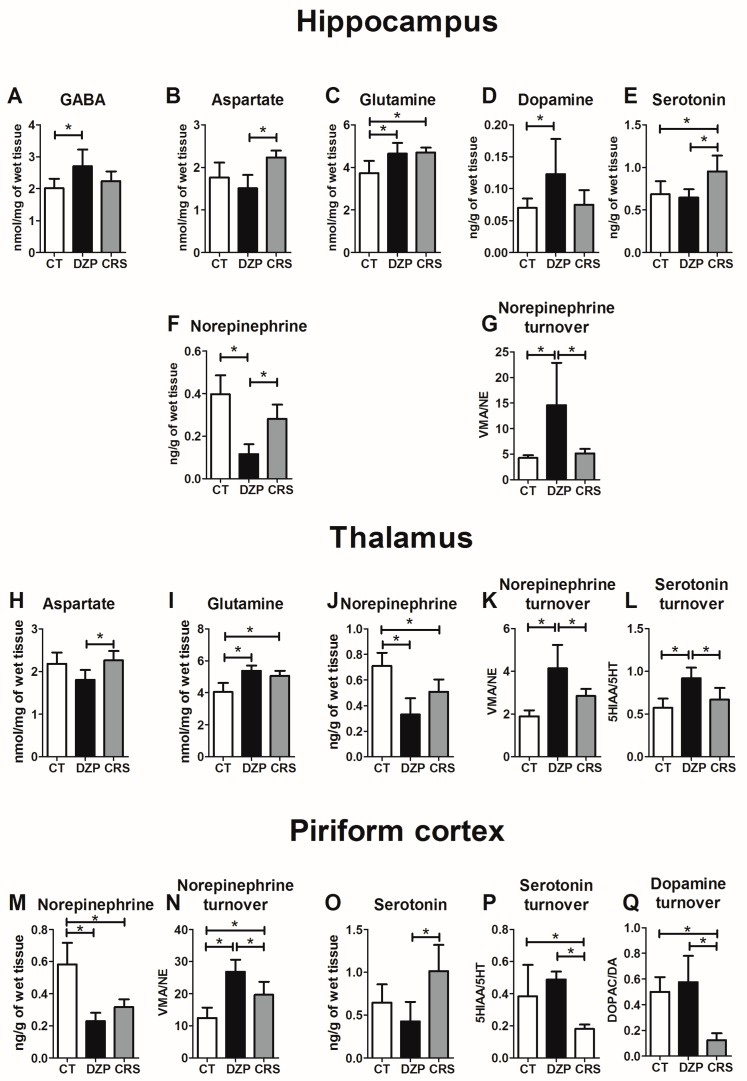
Effect of SE and CRS treatment on monoamine and amino acid levels in the hippocampus, thalamus, and piriform cortex. Only compounds for which significant differences were found are illustrated. * *p* < 0.05: statistically significant difference. No statistical difference was observed in the following analyzes: DA turnover, 5-HT turnover, and GLN, GLY, and TAU in the hippocampus; DA turnover, DA, 5-HT, GLN, GLY, TAU, and GABA on the thalamus; and DA, ASP, GLU, GLN, GLY, TAU, and GABA in the piriform cortex.

Hippocampus: The concentrations of monoamines and amino acids in the hippocampus of rats from control (CT), diazepam (DZP), and CRS groups are shown in Table 1, Figure 2, and Supplementary Figures S2–S4. Our results point to a significant increase in the levels of GABA and glutamine (GLN) in the DZP group as compared to CT rats (Figure 2A,C). The GLN level was increased in the CRS group compared to the CT group, and the aspartate (ASP) level was increased in CRS compared to DZP rats (Figure 2B,C).

Regarding monoamines, norepinephrine (NE) levels in the hippocampus were reduced in DZP compared to CT and CRS rats (Figure 2F), dopamine (DA) levels were increased in DZP compared to CT rats (Figure 2D), and serotonin (5-HT) levels were increased in the CRS compared to CT and DZP groups (Figure 2E). The utilization rate of NE (vanilmandelic acid, (VMA)/NE) was increased in DZP compared to CT and CRS groups (Figure 2G), while the utilization rate of DA (3,4-hydroxyphenylacetic acid (DOPAC)/DA) and 5-HT (5-hydroxyindoleacetic acid (5HIAA)/serotonin (5HT)) was unchanged. 

Thalamus: As shown in Table 1, the level of GLN in DZP and CRS rats was increased as compared to CT rats (Figure 2I). There was also an increase of ASP in CRS compared to DZP rats (Figure 2H). The level of NE was reduced in DZP and CRS in comparison to CT rats (Figure 2J), but no difference was found in the level of DA and 5-HT. The utilization rates of NE and 5-HT were increased in DZP compared to CT and CRS rats (Figure 2K,L), while the utilization rate of DA was unchanged.

Piriform cortex: As summarized in Table 1, there was no intergroup difference regarding the level of amino acids. On the other hand, the level of NE was reduced in DZP and CRS in comparison to CT rats (Figure 2N), while no difference in the level of DA was observed. The level of 5-HT was significantly increased in CRS compared to CT and DZP groups (Figure 2O). Furthermore, NE utilization rate was increased in DZP and CRS compared to CT rats (Figure 2N), while the utilization rate of DA and 5HT (Figure 2P) was greatly reduced in CRS compared to CT and DZP groups (Figure 2Q).

### 2.2. c-Fos Immunostaining

The c-Fos data was normalized to the volume of each structure (number of c-Fos positive cells/volume in mm^3^). Table 2 shows the number of immunopositive cells in the different structures of interest and summarizes statistical results. c-Fos expression in all structures analyzed can be found in Supplementary Figure S5.

**Figure 3:**
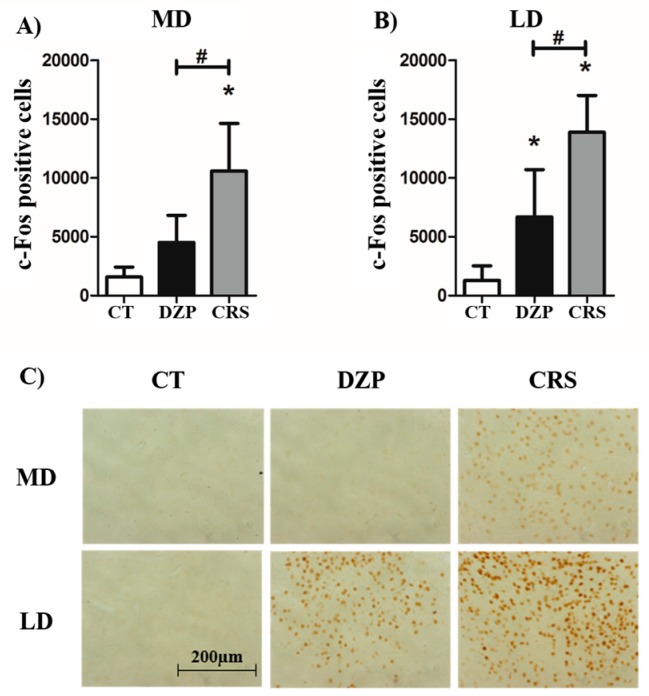
Mean density of c-Fos positive cells normalized to volume for (**A**) MD and (**B**) LD thalamic nuclei; (**C**) c-Fos immunostaining in MD and LD of CT, DZP and CRS rats. * *p* < 0.05: statistically significant difference from CT rats; ^#^ *p* < 0.05: statistically significant difference from DZP rats.

In both groups that underwent SE (CRS or DZP) and in all structures analyzed, c-Fos expression was increased in comparison to CT rats (Supplementary Figure S5), except in the MD where activity was increased only in the CRS over the CT group (Figure 3A). The comparison between CRS and DZP groups showed a significantly larger increase in activation in the MD and LD nuclei in CRS treated rats than in DZP group (Figure 3A,B). Figure 3C shows the c-Fos immunostaining in these areas.

**Figure 4:**
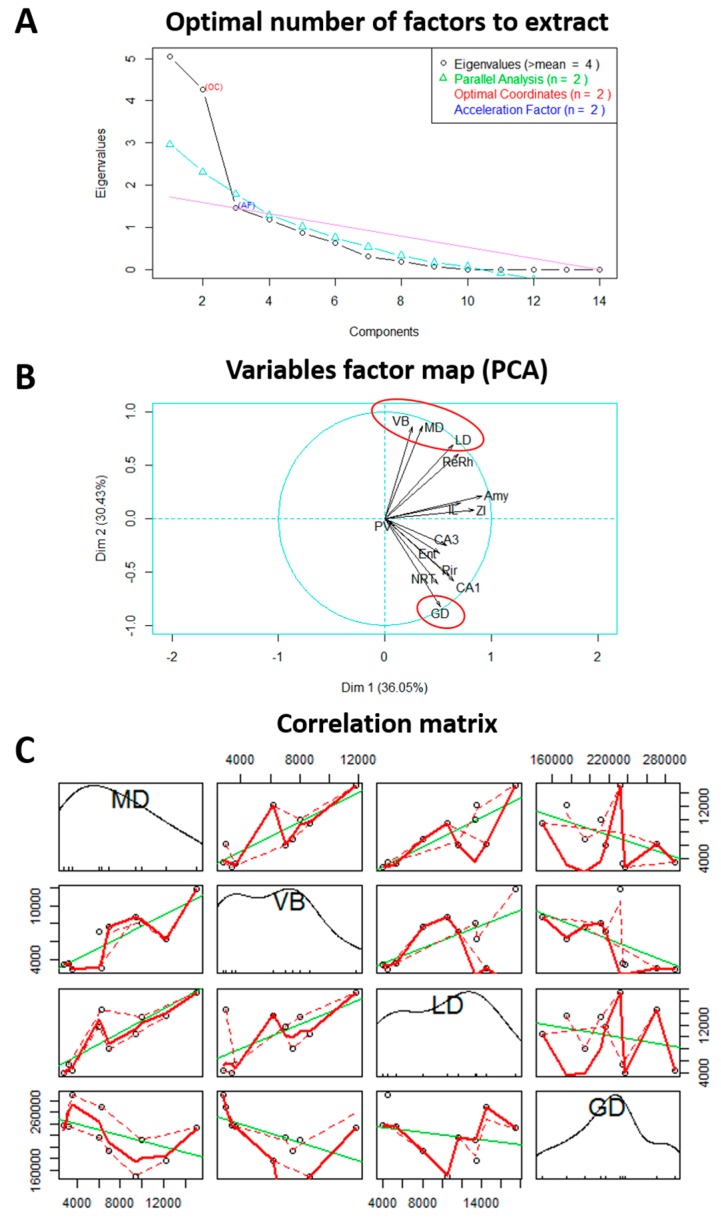
Factorial analysis of brain activity. (**A**) Optimal number of factors to extract; (**B**) Map of the relationship between the different variables mainly showing a positive correlation between ventrobasal (VB), mediodorsal (MD) and laterodorsal (LD) thalamic nuclei, and a negative correlation of these nuclei with dentate gyrus (DG); (**C**) Correlation matrix two-by-two illustrating the correlation between these four structures.

The factorial analysis of the data generated two components and revealed a positive correlation between MD, LD, and ventrobasal (VB) thalamic nuclei, which were negatively correlated with the dentate gyrus (DG) (Figure 4). This result indicate, for example, that a rat with MD activation has simultaneously greater activation of the LD and VB nuclei, and little activation of DG.

## 3. Discussion

In the pilocarpine model, CRS is a molecule capable of producing significant changes in the epileptogenic process. In addition to strong neuroprotection, a subpopulation of rats treated with CRS developed NCS instead of the convulsive seizures commonly observed in this model [10]. NCS, the main feature of absence epilepsy, is characterized by the presence of SWDs, accompanied by behavioral arrest. In rodents these seizures occur at a frequency of 6–10 Hz and are generated within a thalamo-cortical loop [12,19]. 

Interestingly, a significant reduction of epileptiform events was observed at 2–3 h after CRS administration (i.e., at 4 h after SE onset) in most rats subsequently developing NCS [10]. Therefore, our current study looked at neurochemical changes (monoamines and amino acids) and mapped neuronal activity in regions related to the initiation and generalization of convulsive seizures 4 h after LiPilo SE onset. 

### 3.1. Neurochemical Changes

As illustrated in Figure 1, CRS may inhibit voltage-gated sodium channels leading to reduced action potential discharges [13]. CRS also reduces glutamatergic transmission by inhibiting AMPA and NMDA excitatory post-synaptic potentials [14]. Moreover, this compound acts on DA, NE, and 5-HT systems [15]. Interestingly, CRS increases the tonic activation of somatodendritic 5-HT_1A_ serotonergic receptors, thereby enhancing their inhibitory action over pyramidal neurons in the hippocampus [15]. Herein, we assessed the levels of amino acids and monoamines 4 h after SE onset in hippocampus, piriform cortex and thalamus, when changes in cortical electrical activity are habitually recorded [9]. These three structures were chosen in relation to their importance in the genesis and generalization of convulsive seizures [18], and the thalamus was studied because of its critical importance in the occurrence of SWDs [12].

Amino acids: In agreement with previous studies [20], the levels of GABA and GLN were increased 4 h after the LiPilo SE onset in rats treated with DZP. However, while CRS did not seem to affect the level of GLN, it normalized the level of GABA in the hippocampus (compared to CT rats). CRS reduces the glutamatergic transmission by acting on AMPA and NMDA receptors [14], which may reduce the severity of SE as observed in CRS-treated rats. In addition, the reduction of SE severity may also be related to the normalization of GABA level in the CRS group.

Monoamines: In hippocampus, thalamus, and piriform cortex of LiPilo SE (DZP rats), there was a reduction in the NE concentration concomitant with an increase in the utilization rate of NE compared to the CT group. DA level increased only in the hippocampus of DZP compared to CT rats. These results are in agreement with previous studies on the hippocampus of rats subjected to pilocarpine SE [20]. CRS treatment appears to normalize the concentration of monoamines back to control values (except 5-HT in the hippocampus, which was increased over CT and DZP levels). In the piriform cortex and thalamus, the NE level was reduced compared to CT rats, and in the piriform cortex only, 5HT and DA turnover were decreased compared to both CT and DZP rats. The effect of CRS on the monoaminergic system is in agreement with a previous study showing a reduction in the firing rate of neurons in the ventral tegmental area (VTA–DA neurons), locus coeruleus (LC–NE neurons) and dorsal raphe nucleus (DRN–5-HT neurons) when CRS was administered over 14 days [15]. Likewise, the stimulation of 5-HT neurotransmission increases SWDs in animal models of absence epilepsy and reduces the occurrence of convulsive seizures in genetic epilepsy-prone rats, (GEPRs) [21]. These data are in line with the present findings.

Therefore, the fact that CRS can act directly on monoaminergic systems [15] and modulate the level of DA, NE, and 5-HT in the hippocampus, and also in thalamus and piriform cortex (as observed in the present study), may contribute to its neuroprotective and insult-, disease-modifying effects. Indeed, these three regions, which have an established role in the epileptic circuitry regarding the origin and generalization of convulsive seizures, receive monoaminergic projections. Hence, by acting on the monoaminergic system, CRS may modulate the onset, severity and/or generalization of seizures, and it clearly emerges from our data that most of the effects of CRS on monoamine levels and turnover rates appear to normalize the values of saline-treated DZP rats recorded in freely seizing, untreated rats back to the CT values. 

### 3.2. Cerebral Activity

The epileptic circuitry recruited during convulsive seizures involves temporal structures such as hippocampus, amygdala, piriform and entorhinal cortices, but also subcortical structures like the thalamus. These structures have specific roles in the genesis and spread (or generalization) of seizures. Mapping the activity of structures related to convulsive seizures showed that all temporal regions and many thalamic nuclei were significantly more activated in all animals undergoing SE (whatever the treatment, CRS or DZP) compared to CT rats. No difference was observed in the activity of temporal structures—the DG, Cornu Ammonis (CA)1, CA3, amygdala (Amy), piriform cortex (Pir), and entorhinal cortex (Ent)—between the CRS and DZP groups. On the other hand, two thalamic nuclei (MD and LD) showed stronger activation in rats treated with CRS than in those receiving DZP. However, it is important to recall that c-Fos labeling reflects regional activation, but does not indicate whether this activation is excitatory or inhibitory. 

Of note, MD and LD thalamic nuclei exert a modulatory role in the control of both convulsive and non-convulsive seizures, and in epileptogenesis [22,23,24,25,26]. The MD has reciprocal connections with limbic structures, and the thalamus is associated to physiological activity during SE, providing a synchronous epileptiform activity between thalamus and hippocampus [22,27]. Interestingly, in rats with chronic epilepsy, convulsive seizure onset occurs simultaneously in hippocampus and MD, and the electrical stimulation of the MD induces seizures that generalize faster than those induced by hippocampal or amygdalar kindling [22,23]. On the other hand, chemical inhibition of the MD completely blocks convulsive seizures [22,28,29]. However, the seizure-blocking effect of the MD is site-specific because the blockade of convulsive seizures was obtained only when the chemical inhibition target was the central portion of the MD, while no effect was observed when the injection was made in the lateral part of the MD [28]. 

Besides the role of the MD in the genesis, generalization and modulation of convulsive seizures, some studies suggest also an important role of the MD in NCS for SWDs. Indeed, in animal models of absence epilepsy, bilateral lesions of the MD completely abolish SWDs [30]. In contrast, chemical stimulation of the MD with kainic acid induces SWDs (accompanied by behavioral arrest), which spread to other thalamic nuclei, cortex and limbic structures [31].

While the MD plays a clear role in epilepsy, the role of the LD is less clear. In the LiPilo model, both the LD and MD show significantly increased Fluoro-jade B labeling [32], and these nuclei (contrary to hippocampus and amygdala) present a superoxide mechanism of neuronal injury [33]. In the WAG/Rij model of absence epilepsy, a higher level of apoptotic cells and a higher expression of caspase-3 were found in the LD [34]. It is interesting to note that CRS treatment produces dose-dependent neuroprotection of the LD and MD [10]. Furthermore, our results indicate that the activity of the LD and MD are positively correlated with the activity of the VB. The VB thalamic complex is an essential component of the thalamocortical loop generating SWDs [12,35]. 

Therefore, by stimulating the MD, both convulsive and absence-like seizures can be induced, while the inhibition or lesion of this nucleus blocks both seizure types. The increased activity in the MD of rats treated with CRS suggest that this thalamic nucleus may be a key structure, together with the LD and VB, in the epileptogenesis-modifying effect of CRS and in the expression of different seizures types observed in the current study.

## 4. Materials and Methods

### 4.1. General Procedure

Animals: Thirty-four adult male Sprague–Dawley rats (350 ± 30 g) provided by the University of São Paulo were housed under controlled conditions (22 ± 1 °C, 12 h/12 h light/dark cycle, lights on at 7:00 a.m.) with water and food ad libitum. The ethics research committee of the Federal University of São Paulo (CEP N° 2072/11) approved all experiments. Efforts were made to minimize pain or discomfort of animals. The experiments were performed following the principles outlined in the Animal Research: Reporting of In Vivo Experiments (ARRIVE) guidelines and the Basel declaration (http://www.basel-declaration.org). The Replacement, Refinement and Reduction of Animals in research (3R) concept has been considered when planning the experiments. 

SE induction: Rats were injected i.p. with 127 mg/kg lithium chloride. About 18 h later they received methylscopolamine (1 mg/kg s.c., Sigma-Aldrich, Saint Louis, MO, USA) in order to limit the undesirable peripheral effects of pilocarpine. SE was induced 30 min later by the administration of pilocarpine (25 mg/kg s.c., Sigma-Aldrich). The control group received lithium chloride and saline instead of pilocarpine.

Carisbamate treatment: One hour after SE onset, rats were randomly administered with diazepam (DZP, 2.5 mg/kg i.m., Roche, Meylan, France) or CRS (90 mg/kg i.p., Johnson & Johnson Research & Development, L.L.C., Raritan, NJ, USA) dissolved in 45% hydroxypropyl-β-cyclodextrin (Acros Organics, Geel, Belgium). DZP administered at low doses enhanced survival of rats without modifying SE characteristics, as reported in [36] and our own experience. The untreated group received saline instead of CRS. Rats were euthanized 4 h after SE onset. The LiPilo-DZP group represents our reference group for the study of SE only. DZP was used in this short-term experiment because it is the standard treatment of all the animals studied in the long-term.

Experimental groups: Three experimental groups were used: (1) CT—a control group receiving lithium chloride and saline; (2) DZP—rats that underwent SE and were treated with DZP 1 h after SE onset; and (3) CRS—rats that underwent SE and were treated with CRS 1 h after SE onset. 

### 4.2. Measurement of Amino Acid and Monoamine Concentrations

Sample preparation and HPLC assay: Eighteen animals we used in this experiment (*n* = 6/group). Four hours after SE onset animals destined to amino acid and monoamine quantification by HPLC were killed by decapitation and the hippocampus, piriform cortex, and thalamus were dissected out, weighed and stored at −80 °C. The amino acids aspartate (ASP), glutamate (GLU), glutamine (GLN), glycine (GLY), taurine (TAU) and gamma-aminobutyric acid (GABA), and the monoamines dopamine (DA), norepinephrine (NE) and serotonin (5-HT) as well as their respective metabolites (DOPAC, HVA, and 5-HIAA) were quantified. The sample preparation protocol was previously described [20]. 

### 4.3. c-Fos Immunolabelling

Histology: Fifteen animals were used for c-Fos expression determinations (5/group). Four hours after SE all rats were deeply anesthetized with a solution of ketamine (80 mg/kg, i.p.)/xylazine (30 mg/kg, i.p.) and transcardially perfused with saline (0.9%) followed by phosphate-buffered 4% formaldehyde. After perfusion, animals were decapitated and brains were kept “in situ”, immersed in the fixative solution for 20 h, removed from the skull, and post-fixed overnight. The brains were sectioned in free-floating coronal slices, 40 µm thick, using a vibratome. Sections were stored in wells containing cryoprotectant at −20 °C. One of the 10 sets of sections between Bregma +3.72 mm and −6.84 mm was used for c-Fos immunolabeling. The sections were incubated with the primary polyclonal anti-c-Fos antibody. After washing, the sections were incubated with the biotinylated secondary antibody. The positive staining was visualized with a solution of 3,3′-diaminobenzidine. The sections were separated and mounted on slides. The specificity of the antibody was tested by incubation without the primary antibody while all other procedures were identical.

Regions of interest delineation: The regions of interest (ROI) were hippocampal subregions (DG, CA1, and CA3), the basolateral amygdaloid nucleus (Amy), the piriform cortex (Pir), the entorhinal cortex (Ent) and thalamic nuclei: paraventricular (PV), ventrobasal (VB), zona incerta (ZI), intermediolateral (IM), thalamic reticular nucleus (TRN), reuniens/rhomboid (ReRh), mediodorsal (MD), and laterodorsal (LD). All regions of interest were delineated based on the Paxinos and Watson rat brain atlas [37]. Supplementary Figure 1 shows the delineation of ROIs.

Stereological design: Estimation of c-Fos positive cells and the volume of regions of interest was obtained using the Cavalieri’s method combined with systematic random sampling [38]. The precision of estimation is described by the coefficient of error. Estimations were performed using the Mercator software and a microscope (Leica DM5500B). Slides were analyzed using a 2.5 X objective. Digital live microscope images were visualized by a high-resolution Microfire by an Optronic camera. For hippocampus, amygdala, piriform and entorhinal cortices, we used a 30 × 30 µm dissector spaced 100 × 100 µm, and for thalamic nuclei we used a 40 × 40 µm dissector spaced 60 × 60 µm. 

Statistical analysis: For c-Fos labeling and neurochemical quantification we used a one-way analysis of variance (ANOVA). The post-hoc Tukey test was used when appropriate to identify statistical significance. Both ANOVA and post-hoc Tukey were performed with 10,000 bootstrap resampling. A factorial analysis was used to assess the correlation between the activities of the different structures of interest labeled with c-Fos. Statistical analysis was performed in R version 3.0.2 [39]. 

## 5. Conclusions

**Figure 5:**
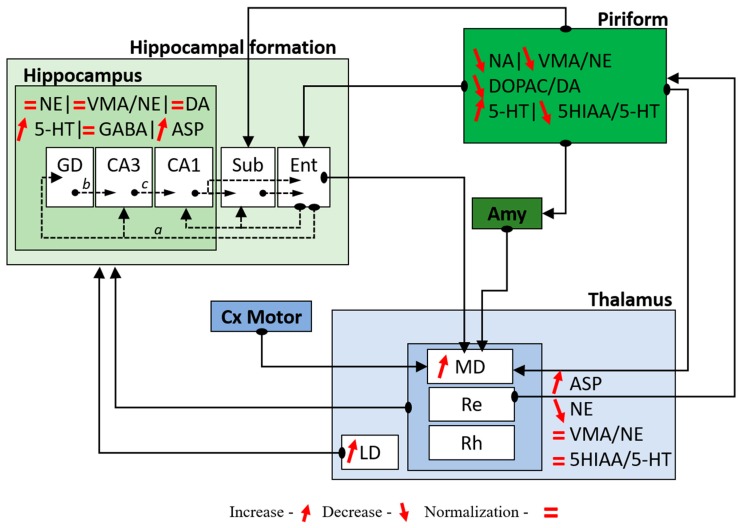
Schematic representation of the acute changes in the hippocampus, piriform cortex, and thalamus based on c-Fos and HPLC-measured changes. Entorhinal cortex (Ent) is the main entrance of hippocampus projecting directly into DG and CA3 via the perforant pathway (a), but projects also to CA1 and the subiculum (Sub). Mossy fibers project from DG to CA3 (b) and the Schaffer collaterals connect CA3 to CA1 (c). CA1 sends efferent projections to the subiculum and the entorhinal cortex. The subiculum sends also efferent projections into entorhinal cortex. The piriform cortex sends projections to the subiculum, entorhinal cortex, amygdala and MD thalamic nucleus, and receives efferent projections coming from the thalamic reuniens nucleus. Finally, the MD also receives afferences coming from entorhinal cortex, amygdala, and motor cortex. Midline thalamic nuclei (MD, Re and Rh) and the LD nucleus send projections to the hippocampal formation. At the bottom of the figure, the comparisons indicated are as follows: increase and decrease occurring in CRS-treated rats compared to CT. Normalization means values identical in CRS and CT rats and most often represent decreases that occur in CRS compared to DZP rats.

Herein, we observed that CRS may affect neuronal excitability during SE. CRS plays a modulatory role on the monoaminergic system, most often preventing the SE-induced changes recorded in DZP rats and potentially minimizing its effect on the genesis and generalization of convulsive seizures. In addition, CRS appears to normalize the concentration of GABA. However, in terms of cellular activity, only the activity of thalamic MD and LD were significantly different in rats treated with CRS compared to animals treated with DZP. Together with literature data, our results suggest that the thalamic MD may be a key region in the modulation of epileptogenesis by CRS, minimizing the changes in that structure, and hence allowing the expression of SWDs instead of convulsive seizures. This hypothesis would need in vivo electrophysiological recording experiments for confirmation. Figure 5 summarizes the connectivity between structures related to convulsive seizures and includes our results in a general functional scheme.

## Figures and Tables

**Table 1:** Effect of status epilepticus (SE) and CRS treatment on the monoamine and amino acid levels in the hippocampus, thalamus, and piriform cortex.

		**Hippocampus**					**Thalamus**					**Piriform Cortex**				
		**CT**	**DZP**	**CRS**	**ANOVA**	**BS**	**CT**	**DZP**	**CRS**	**ANOVA**	**BS**	**CT**	**DZP**	**CRS**	**ANOVA**	**BS**
**Monoamines (ng/g)**	NE	0.39	0.12 *	0.28 ^#^	F_(2,15)_ = 20.73; *p* < 0.001	*p* < 0.001	0.71	0.33 *	0.51 *	F_(2,15)_ = 19.76; *p* < 0.001	*p* < 0.001	0.58	0.23 *	0.32 *	F_(2,15)_ = 35.88; *p* < 0.001	*p* < 0.001
	VMA	1.64	1.43	1.41	F_(2,15)_ = 1.64; *p* = 0.226	*p* = 0.269	1.32	1.27	1.42	F_(2,15)_ = 0.99; *p* = 0.392	*p* = 0.307	7.02	6.08	6.03	F_(2,15)_ = 2.67; *p* = 0.101	*p* = 0.160
	VMA/NE	4.27	14.58 *	5.16 ^#^	F_(2,15)_ = 9.33; *p* = 0.002	*p* = 0.002	1.90	4.14 ^*^	2.84 ^#^	F_(2,15)_ = 18.07; *p* < 0.001	*p* < 0.001	12.40	26.82 *	19.67 *^#^	F_(2,15)_ = 21.03; *p* < 0.001	*p* < 0.001
	DA	0.07	0.12	0.07	F_(2,15)_ = 4.31; *p* = 0.033	*p* = 0.087	0.05	0.09	0.06	F_(2,15)_ =2.35; *p* = 0.129	*p* = 0.172	0.15	0.13	0.23	F_(2,15)_ = 3.73; *p* = 0.048	*p* = 0.068
	DOPAC	0.01	0.02	0.02	F_(2,15)_ = 0.67; *p* = 0.521	*p* = 0.383	0.01	0.03	0.01	F_(2,15)_ = 3.19; *p* = 0.069	*p* = 0.128	0.07	0.07	0.03 *^#^	F_(2,15)_ = 15.12; *p* < 0.001	*p* = 0.011
	DOPAC/DA	0.23	0.17	0.24	F_(2,15)_ = 0.74; *p* = 0.490	*p* = 0.311	0.06	0.29	0.15	F_(2,15)_ = 2.59; *p* = 0.107	*p* = 0.137	0.50	0.57 *	0.12 *	F_(2,15)_ = 23.35; *p* <0 .001	*p* < 0.001
	HVA	0.07	0.08	0.08	F_(2,15)_ = 0.06; *p* = 0.941	*p* = 0.492	0.05	0.09	0.06	F_(2,15)_ = 1.44; *p* = 0.267	*p* = 0.263	0.05	0.04	0.16	F_(2,15)_ = 0.71; *p* = 0.504	*p* = 0.340
	HVA/DA	1.04	0.66	1.06	F_(2,15)_ = 3.78; *p* = 0.046	*p* = 0.051	1.53	0.88	1.23	F_(2,15)_ = 1.45; *p* = 0.264	*p* = 0.227	0.73	0.55	1.51 ^#^	F_(2,15)_ = 7.08; *p* < 0.007	*p* < 0.01
	5-HT	0.68	0.65	0.95 *^#^	F_(2,15)_ = 8.50; *p* = 0.003	*p* = 0.007	1.13	1.12	1.29	F_(2,15)_ = 1.94; *p* = 0.177	*p* = 0.172	0.65	0.43	1.01 ^#^	F_(2,15)_ = 8.58; *p* = 0.003	*p* = 0.011
	5HIAA	0.30	0.51 *	0.48 *	F_(2,15)_ = 11.55; *p* < 0.001	*p* < 0.001	0.64	1.04 *	0.84	F_(2,15)_ = 13.16; *p* < 0.001	*p* < 0.001	0.23	0.26	0.18	F_(2,15)_ = 4.28; *p* = 0.033	*p* = 0.077
	5HIAA/5-HT	4.76	7.36	6.54	F_(2,15)_ = 3.09; *p* = 0.075	*p* = 0.136	0.57	0.92 *	0.67 ^#^	F_(2,15)_ = 13.83; *p* < 0.001	*p* < 0.001	0.38	0.49	0.18 *^#^	F_(2,15)_ = 18.61; *p* < 0.001	*p* < 0.001
		**Hippocampus**					**Thalamus**									
		**CT**	**DZP**	**CRS**	**ANOVA**	**BS**	**CT**	**DZP**	**CRS**	**ANOVA**	**BS**					
**Amino acids (nmol/mg)**	ASP	1.77	1.51	2.23 ^#^	F_(2,15)_ = 8.59; *p* = 0.003	*p* = 0.005	2.18	1.80	2.26 ^#^	F_(2,15)_ = 6.93; *p* = 0.007	*p* = 0.013					
	GLU	8.42	8.37	9.11	F_(2,15)_ = 0.51; *p* = 0.608	*p* = 0.382	8.89	8.57	8.22	F_(2,15)_ = 0.35; *p* = 0.705	*p* = 0.398					
	GLN	3.63	4.65 *	4.67 *	F_(2,15)_ = 9.94; *p* < 0.001	*p* = 0.002	4.04	5.37 *	5.06 *	F_(2,15)_ = 17.57; *p* < 0.001	*p* < 0.001					
	GLY	0.38	0.44	0.44	F_(2,15)_ = 1.00; *p* = 0.388	*p* = 0.288	0.48	0.50	0.49	F_(2,15)_ = 0.07; *p* = 0.932	*p* = 0.932					
	TAU	5.39	4.98	5.55	F_(2,15)_ = 0.78; *p* = 0.471	*p* = 0.311	2.36	2.15	2.38	F_(2,15)_ = 1.11; *p* = 0.354	*p* = 0.292					
	GABA	2.00	2.70 *	2.24	F_(2,15)_ = 4.99; *p* = 0.021	*p* = 0.045	2.55	2.51	2.23	F_(2,15)_ = 1.90; *p* = 0.182	*p* = 0.156					

One-way ANOVA and Tukey post-hoc test with 10,000 bootstrap (BS) resampling of amino acids and monoamines in the hippocampus, thalamus, and piriform cortex. Abbreviations: VMA—vanilmandelic acid; NE—norepinephrine; DOPAC—3,4-hydroxyphenylacetic acid; DA—dopamine; 5-HIAA—5-hydroxyindoleacetic acid; HVA—homovanillic acid; 5-HT—serotonin; ASP—aspartate; GLU—glutamate; GLN—glutamine; GLY—glycine; TAU—taurine; GABA—gamma-aminobutyric acid. * *p* < 0.05: statistically significant difference from the CT group; ^#^ *p* < 0.05: statistically significantly difference from the diazepam (DZP) group.

**Table 2:** Quantification of c-Fos positive cells in CT, DZP and CRS rats.

Structure	Number of Cells			ANOVA	Bootstrap
	CT	DZP	CRS		
PV	6471	37,435 *	43,927 *	F_(2,12)_ = 77.17; *p* < 0.001	*p* < 0.001
IL	1275	26,498 *	28,314 *	F_(2,12)_ = 119.88; *p* < 0.001	*p* < 0.001
ReRh	995	21,466 *	27,867 *	F_(2,12)_ = 73.00; *p* < 0.001	*p* < 0.001
MD	1571	4517	10,588 *^#^	F_(2,12)_ = 21.85; *p* < 0.001	*p* < 0.001
RTN	880	29,169 *	25,824 *	F_(2,12)_ = 52.09; *p* < 0.001	*p* < 0.001
ZI	1537	11,858 *	13,409 *	F_(2,12)_ = 6.08; *p* = 0.015	*p* = 0.009
VB	852	4866 *	7562 *	F_(2,12)_ = 10.59; *p* = 0.002	*p* = 0.003
LD	1291	6698 *	13,880 *^#^	F_(2,12)_ = 33.66; *p* < 0.001	*p* < 0.001
GD	2865	234,694 *	208,044 *	F_(2,12)_ = 68.35; *p* < 0.001	*p* < 0.001
CA3	5832	79,664 *	80,145 *	F_(2,12)_ = 48.48; *p* < 0.001	*p* < 0.001
CA1	2724	130,109 *	123,864 *	F_(2,12)_ = 31.19; *p* < 0.001	*p* < 0.001
Pir	3778	213,143 *	203,401 *	F_(2,12)_ = 70.09; *p* < 0.001	*p* < 0.001
Ent	5117	153,586 *	136,315 *	F_(2,12)_ = 47.08; *p* < 0.001	*p* < 0.001
Amy	4097	74,388 *	93,834 *	F_(2,12)_ = 18.78; *p* < 0.001	*p* < 0.001

One-way ANOVA and Tukey post-hoc test with 10,000 bootstrap resampling of the number of c-Fos-positive cells. Abbreviations: PV—paraventricular thalamic nucleus; IL—intralaminar thalamic nucleus; ReRh—reuniens/ rhomboid thalamic nucleus; MD—mediodorsal thalamic nucleus; RTN—reticular thalamic nucleus; ZI—zona incerta; VB—ventro-basal thalamic nucleus; LD—laterodorsal thalamic nucleus; DG—dentate gyrus; CA3—Cornu Ammonis 3; CA1—Cornu Ammonis 1; Pir—piriform cortex; Ent—entorhinal cortex; Amy—amygdala. * *p* < 0.05: statistically significant difference from the CT group; ^#^ *p* < 0.05: statistically significant difference from DZP group.

## Supplementary Materials

The following are available online at www.mdpi.com/1424-8247/10/4/85/s1, Figure S1: Regions of interest (ROI) delineated on sections taken from the Paxinos and Watson atlas (1998); Figure S2: Hippocampal level of amino acids (nmol/mg) and mono amines (ng/g); Figure S3: Thalamic level of amino acids (nmol/mg) and mono amines (ng/g); Figure S4: Level of amino acids (nmol/mg) and mono amines (ng/g) in the Piriform cortex; and Figure S5: Neuronal activity in thalamic nuclei and temporal regions. c-Fos positive cells, normalized by volume.
