# Supplementary material for: Neurochemical Changes and c-Fos Mapping in the Brain after Carisbamate Treatment of Rats Subjected to Lithium–Pilocarpine-Induced Status Epilepticus
Source: Pharmaceuticals (Basel). 2017 Nov 1;10(4):85. doi: 10.3390/ph10040085 (PMC5748642; doi:10.3390/ph10040085)
Supplement: Supplementary file 1 [file pharmaceuticals-10-00085-s001.zip › Supp data.docx]

| 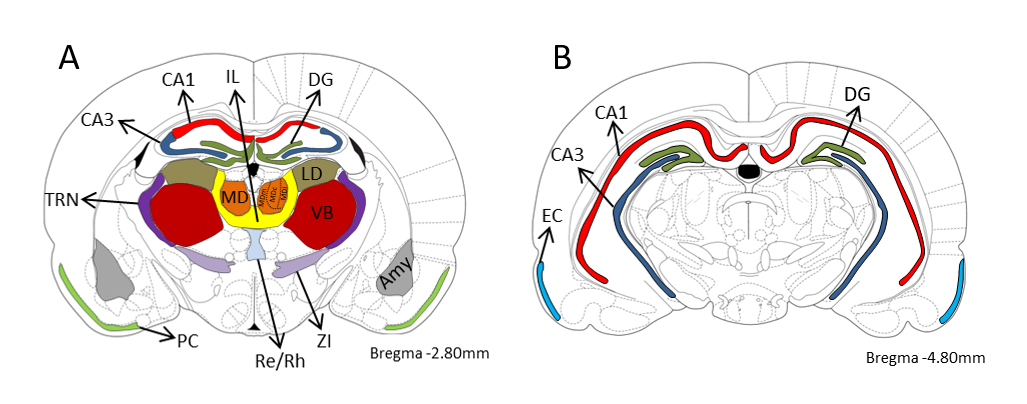 |  |
| --- | --- |

**Figure S1.** Regions of interest (ROI) delineated on sections taken from the Paxinos and Watson atlas (1998). A) Bregma -2.80 showing MD, LD, IL, VB, TRN, ZI, Re/Rh nuclei of thalamus, basolateral amygdala, PC and ventral subfields of hippocampus CA1, CA3 and DG. B) Bregma -4.80 showing EC and dorsal subfields of hippocampus CA1, CA3 and DG.


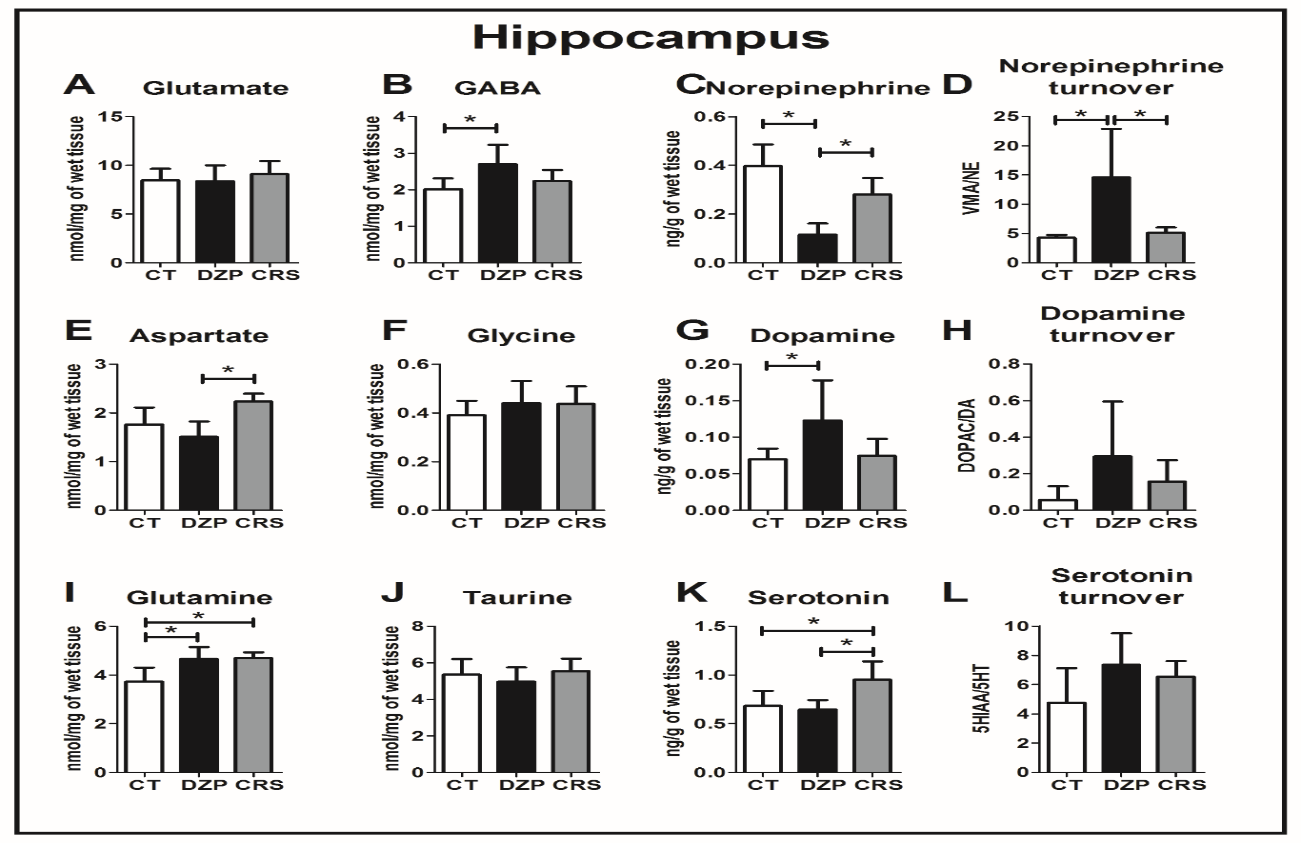


**Figure S2**: Hippocampal level of amino acids (nmol/mg) and mono amines (ng/g).


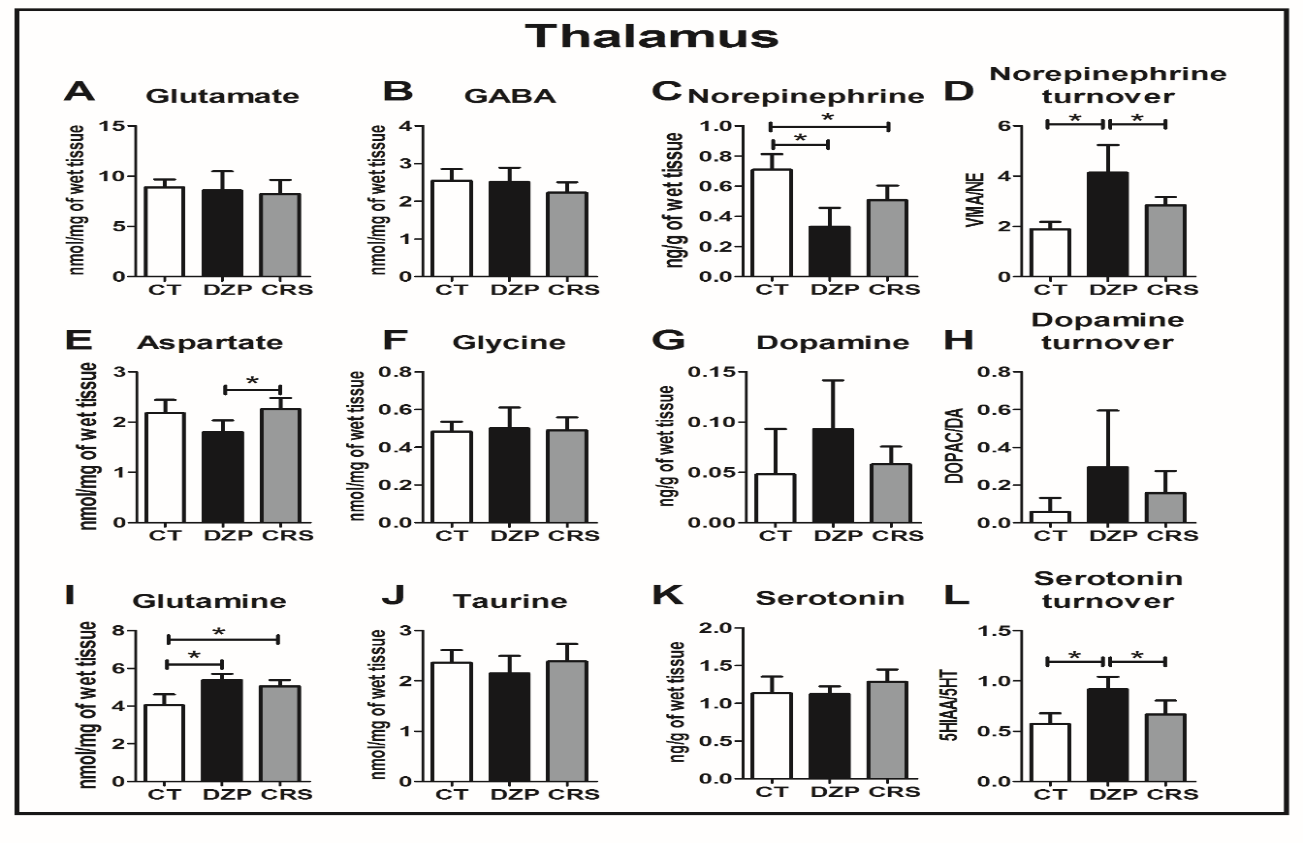


**Figure S3**: Thalamic level of amino acids (nmol/mg) and mono amines (ng/g).


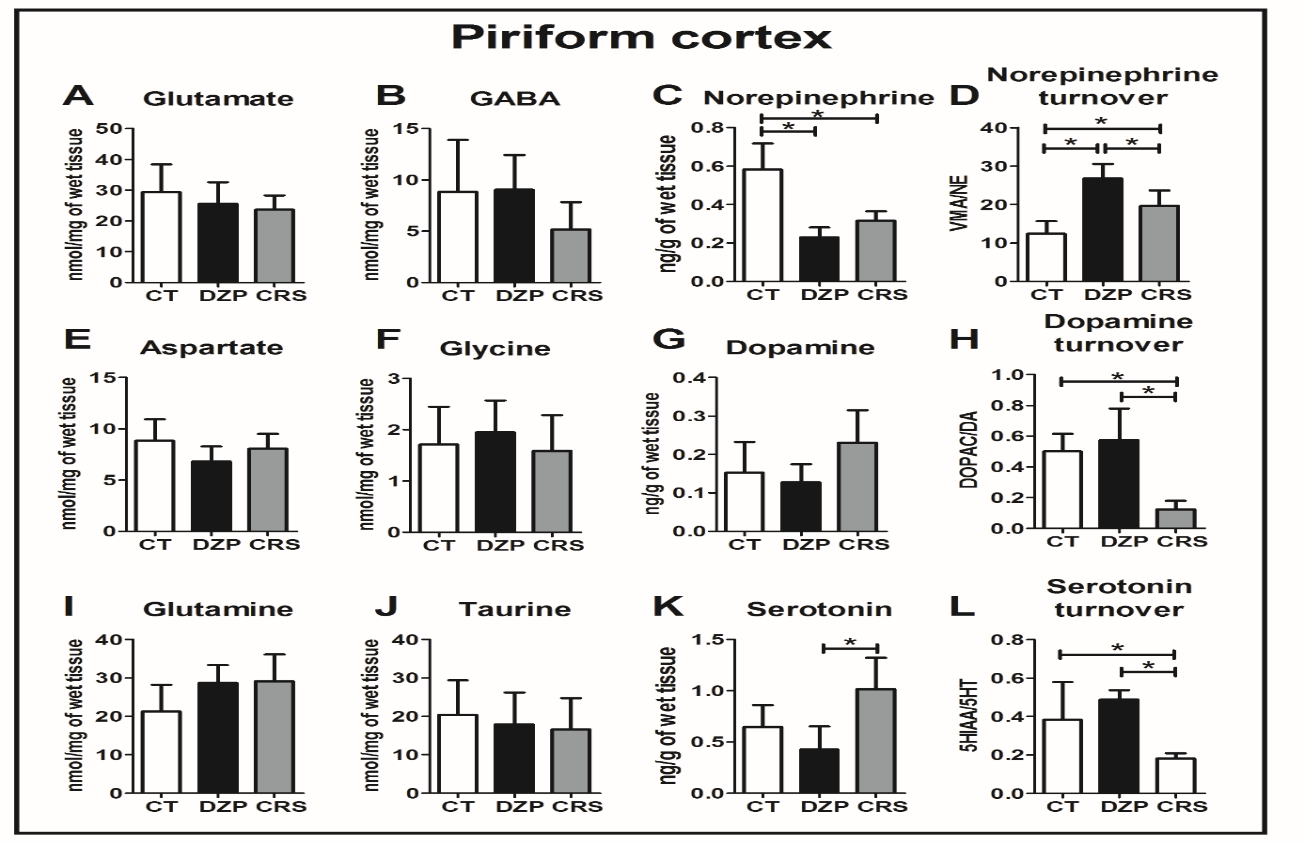


**Figure S4**: Level of amino acids (nmol/mg) and mono amines (ng/g) in the Piriform cortex.


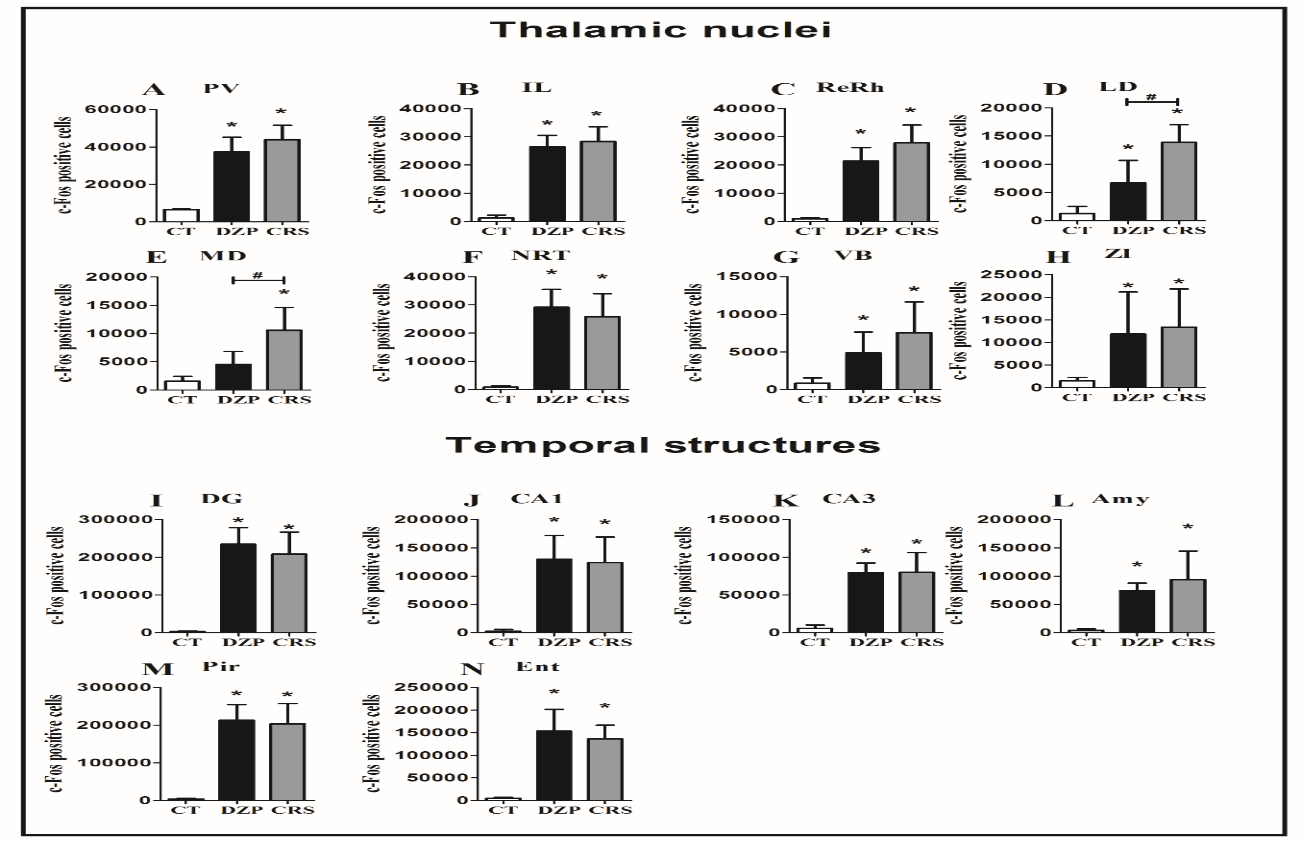


**Figure S5**: Neuronal activity in thalamic nuclei and temporal regions. c-Fos positive cells, normalized by volume. PV - paraventricular; IL - intralaminar; ReRh - reuniens/ rhomboid; MD – mediodorsal ; RTN – reticular thalamic nucleus ; ZI - zona incerta; VB - ventro-basal; LD - laterodorsal; DG – dentate gyrus; CA3 - cornu ammonis 3; CA1 - cornu ammonis 1; Pir –piriform cortex; Ent - entorhinal cortex; Amy - amygdala.
